# La Grippe

**Published:** 1910-04

**Authors:** E. P. Green

**Affiliations:** Hickory Flat, Ala.


### LA GRIPPE.

BY E. P. GREEN, M. D., HICKORY FLAT, ALA.

I consider the subject of vast importance because so many people suffer on account of this painful disease, LaGrippe.

LaGrippe, or epidemic catarrhal fever is an acute infectious disease characterized by pain and inflammations of the mucous membrane accompanied by fever and a tendency to nervous prostration.

*Etiology*:—LaGrippe usually begins in the East and spreads westward with great rapidity. Epidemics usually occur in the winter, attacking all ages and sexes alike. One attack does not produce immunity, but seems to favor subsequent attacks. The cause of the disease is the influenza bacillus which probably reaches the organism through the respiratory tract. The bacillus was discovered by Peiffer in 1892.

The incubation period is usually from one to four days. The onset usually sudden, beginning with a chill or chilly sensations, a severe headache, sometimes vomiting, aching of the limbs, loss of appetite, sometimes a paroxysmal cough, pain in the chest, and catarrhal symptoms. The temperature rises rapidly to from 101 to 104 F., according to severity of attack. In the gastro-intestinal type the patient has a heavily coated tongue, there are persistent vomiting and diarrhoea, with pain and tenderness over th abdomen, sometimes so severe as to suggest appendicitis.

Pneumonia is far the most common and fatal complication. It may be either broncho or lobar, but is most frequently broncho. The expectoration is like that of bronchitis, rarely ever being "rusty." The temperature is lower than primary pneumonia and is often remittent, though the duration is longer and heart failure is more frequent. Some of the rarer complications are pleurisy, pericarditis, nephritis, myocarditis, functional cardiac disorders, such as palpitation and irregular heart action, which may cause sudden death. In the nervous type the complications are meningitis; encephalitis, cerebral abscess, paralysis, myelitis and neuritis. Some mental disorders occur, such as melancholia and dementia. The diagnosis is usually easy during an epidemic. The disease is rarely fatal except when complicated with pneumonia or in the aged or debilitated or in patients with advanced cardiac, pulmonary or nephritic lesions.

As to treatment, the patient should be kept indoors and isolated if possible and on liquid diet. The room should be kept an even temperature from 70 to 80 degrees Farh. Give a mercurial purge at the onset followed by salts if necessary. Much can be done to relieve the nasal and throat symptoms by the use of a spray of menthol and eucalyptol in liquid aboline. It is a good idea to keep the emunctories of the body open so as to eliminate as much of the toxins as possible. For the relief of pain many physicians object to the use of the coal tar preparations. I treat my patients according to the individual case. In using the coal tar preparations I believe in supplementing them with stimulants if necessary. Acetanilid with caffeine, aspirin and antipyrin all work well. While the coal tar preparations are good I think there are other remedies that are good. I think it is best to leave off the opiates as long as possible on account of locking up the secretions, but if necessary I give them and follow with purgatives, mustard plasters will work well in giving relief from local pains; as to expectorants carbonate of ammonia is a good one, if it is not too irritating to the stomach, if so, then the muriate should be used. We must be governed according to age, severity of the attack, and the general condition of the patient. During convalescence tonics should be administered to build up the system and if much exhausted the patients should not return to work too early.

---

The president of the American Gynecological Society has appointed a Committee to report at the next annual meeting in Washington, on the Present Status of Obstetrical Teaching in Europe and America, and to recommend improvements in the scope and character of the teaching of Obstetrics in America.

The committee consists of the Professors of Obstetrics in Columbia University, University of Pennsylvania, Harvard, Jefferson Medical College, Johns Hopkins University, Cornell University and the University of Chicago.

Communications from anyone interested in the subject will be gladly received by the chairman of the Committee, Dr. B. C. Hirst, 1821 Spruce St., Philadelphia, Pa.

